# Exertional Near-Syncope: Pericardial Cyst as a Cause of Left Ventricular Outflow Obstruction

**DOI:** 10.5811/cpcem2022.6.56749

**Published:** 2022-08-08

**Authors:** Ryan Offman, Joseph Skopek

**Affiliations:** Michigan State University College of Osteopathic Medicine, Mercy Health – Muskegon, Department of Emergency Medicine, Muskegon, Michigan

**Keywords:** pericardial cyst, near-syncope

## Abstract

**Case Presentation:**

A 41-year-old otherwise healthy male presented to the emergency department with recurrent exertional near-syncope. He was eventually found to have a large pericardial cyst causing an outflow obstruction. After resection of the cyst by cardiothoracic surgery, he had an uneventful hospital course and was discharged seven days later with no recurrent syncopal episodes.

**Discussion:**

We describe an otherwise healthy patient who exhibited symptomatic left ventricular outflow obstruction from a pericardial cyst. These cysts are usually benign and asymptomatic, although they can progress to cause significant morbidity or mortality. Surveillance is recommended if no hemodynamic compromise is present. Patients who are symptomatic or have hemodynamic compromise may undergo needle aspiration or thoracoscopy with resection.

## CASE PRESENTATION

**Image 1:**
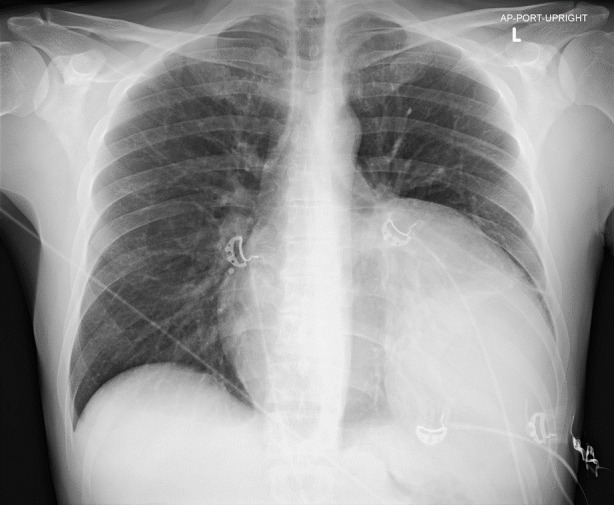
A plain film radiograph of the chest revealing cardiomegaly with a left lower lobe opacity versus mass outlined by arrows.

A 41-year-old male presented to the emergency department (ED) describing recurrent, exertional presyncope. While using a sitz bath, he became diaphoretic, short of breath, and experienced one to two minutes of chest pain and lightheadedness. Subsequently, the patient noted he would have recurrence of these symptoms with minimal exertion. Symptoms would resolve with rest. He denied history of venous thromboembolism, prior exertional chest pain, or any significant rectal bleed. In the ED, he was hemodynamically stable. Physical examination demonstrated decreased left-sided breath sounds but was otherwise normal. A plain film of his chest revealed cardiomegaly vs underlying mass (Image 1). A computed tomography angiography of his chest was obtained.

**Image 2:**
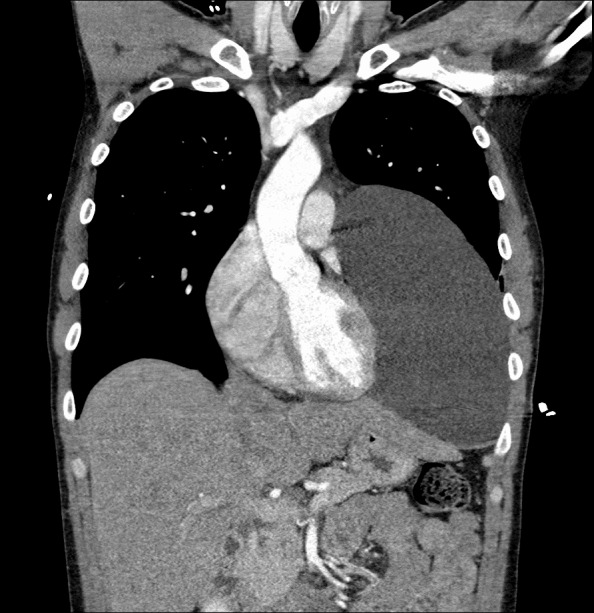
A computed tomography angiogram revealing a large cystic lesion in the left lower and mid-chest abutting the mediastinum and left heart border measuring 20 × 11 × 17 centimeters. The arrows outline the mass.

**Image 3:**
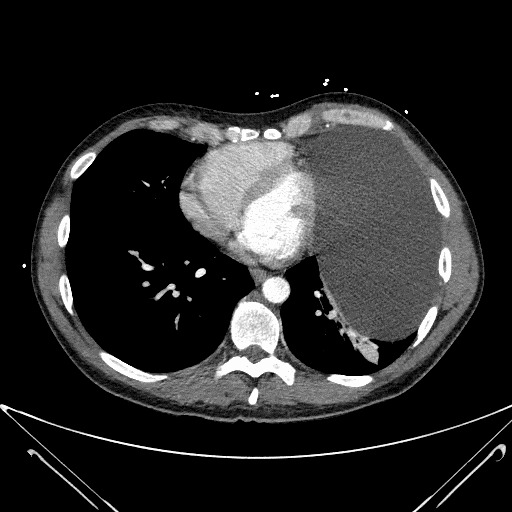
A computed tomography angiogram revealing the large pericardial cyst in an axial cut outlined by the arrows.

Images revealed a large pericardial cyst (Images 2 and 3). An emergent cardiology-performed echocardiogram was performed showing the compression of the inferior left ventricle. High sensitivity troponin and D-dimer were both negative. He was admitted to the hospital and his pericardial cyst was resected by cardiothoracic surgery the following day. Histopathology demonstrated a benign thymic mass. He had an uneventful hospital course and routine follow-up without return of his shortness of breath or presyncopal episodes.

## DISCUSSION

Pericardial cysts are generally asymptomatic, benign masses. However, they occasionally can cause chest pain and/or shortness of breath. They typically form at the cardiophrenic angle.^1^ They are usually asymptomatic but can occasionally cause cardiac tamponade or outflow obstruction. Echocardiography is a key diagnostic imaging modality to determine management in these cases. Management typically involves serial echocardiograms if the cycsts are asymptomatic.^2^ Treatment options consist of needle aspiration or thoracoscopy with resection depending on the size of the cyst and symptoms resulting from the cyst. The most worrisome complications of pericardial cysts include cardiac tamponade, heart failure, atrial fibrillation, outflow obstruction, and airway collapse.^3^ Our patient demonstrated compression of the inferior wall of the left ventricle, which could potentially progress to sudden cardiac death by pericardial tamponade.^4^

Pericardial cysts and other mediastinal masses are important considerations in the differential diagnosis of syncope to clinicians, and they may not be able to be differentiated on radiograph alone. The cysts can manifest with similar clinical presentations as more common etiologies. Transthoracic echocardiogram may be indicated during the emergent work-up of these patients especially if they present with exertional symptoms, unstable vital signs, or abnormal chest radiography.

**CPC-EM Capsule**

What do we already know about this clinical entity?

*Pericardial cysts are usually asymptomatic and benign but can occasionally cause cardiac tamponade or outflow obstruction*.

What is the major impact of the image(s)?

*Pericardial cysts are important considerations in the differential diagnosis of syncope to clinicians, and they may not be able to be differentiated on radiograph alone*.

How might this improve emergency medicine practice?

*Cardiomegaly on plain radiograph has a broad differential and echocardiography is key in assessing cardiovascular effects of pericardial cysts*.
